# Determinants of facility based–deliveries among urban slum dwellers of Kampala, Uganda

**DOI:** 10.1371/journal.pone.0214995

**Published:** 2019-04-18

**Authors:** Lestine Bitakwitse Atusiimire, Peter Waiswa, Lynn Atuyambe, Victoria Nankabirwa, Monica Okuga

**Affiliations:** 1 Makerere University, College of Health Sciences, School of Public Health, Kampala, Uganda; 2 Centre of Excellence for Maternal and Newborn Health, Makerere University College of Health Sciences School of Public Health, Kampala, Uganda; 3 Department of Health Policy, Planning and Management, Makerere University, College of Health Sciences, School of Public Health, Kampala, Uganda; 4 Department of Community Health and Behavioral Sciences, Makerere University, College of Health Sciences, School of Public Health, Kampala, Uganda; 5 Department of Epidemiology and Biostatistics Makerere University, College of Health Sciences, School of Public Health, Kampala, Uganda; Harvard Medical School, UNITED STATES

## Abstract

**Background:**

Delivery in health facilities is a proxy for skilled birth attendance, which is an important intervention to reduce maternal and neonatal mortality. We investigated the determinants of facility based deliveries among women in urban slums of Kampala city, Uganda.

**Methods:**

A cross sectional study using quantitative methods was used. A total of 420 mothers who had delivered in the past one year preceding the survey, were randomly selected and interviewed using a pre-tested interviewer administered questionnaire. Univariate and multivariable logistic regression analysis was done to determine independent predictors of facility based deliveries.

**Results:**

Ninety-five percent of respondents attended at least one antenatal care visit and 66.1%delivered in a health facility. Independent predictors of health facility births included exposure to media concerning facility delivery (OR = 2.5, 95% CI = 1.6–3.9), ANC attendance less than 4 times (OR = 0.6, 95% CI = 0.3–0.9) and timing of first ANC visit in the 2 and 3^rd^ trimesters of pregnancy (OR = 0.5 95% CI = 0.3–0.8).

**Conclusion:**

Despite good physical access, a third of mothers did not deliver in health facilities. Increasing health facility births among the slum dwellers can be improved through interventions geared at increased awareness, starting ANC in early stages of pregnancy and attending at least 4 ANC visits.

## Introduction

Despite various national and international initiatives to improve maternal health, 303,000 women died of pregnancy related causes worldwide in 2015, with 99% of these deaths occurring in low and middle income countries[1]. In Sub-Saharan Africa, a woman’s risk of dying from complications of pregnancy and childbirth over the course of her lifetime is 1 in 160, compared to 1 in 3,700 in high income countries [2–4]. These same regions account for, 98% of the approximately 3.3 million global neonatal deaths that occur annually. Also around 50million birth are not attended by trained healthcare personnel [5] and this lack of professional assistance is considered as one of the major factors in maternal and infant mortality in these regions[6, 7].Home delivery, poverty and fewer ANC consultations are the most prominent risk factors for still birth, early neonatal and perinatal deaths, among urban poor as most of them die at home under the care of mothers, relatives, and traditional birth attendants [8–12].

In Uganda, the maternal mortality ratio is estimated to be 336/100,000live births [13]. And at the moment, Uganda is working to meet the WHO recommendation of having skilled attendance for all births. According to the 2016 Ugandan Demographic and Health Survey (UDHS) report, 95% women at least receive one antenatal care, 59% women are assisted by skilled personnel at delivery and 57% women had health facility delivery [13]. Despite the increase in facility births, it is noted that there is continued high maternal-mortality ratio, with substantial variation across Ugandan regions. The use of reproductive health services remains low and home delivery among women of child bearing age is widespread [14–16]. This is evidenced in other resource-poor settings, where home delivery is usually the cheapest option; though it is associated with attendant risks of infection and lack of available equipment should complications occur[17].

In urban slums, the poor are a highly vulnerable and marginalized group with un planned/poor housing, no basic amenities and low use of skilled care at delivery[18]. Morbidity in urban poor populations is also influenced by social determinants such as social gradient, social exclusion, social support, stress, and physical activity; and suboptimal health behaviors[19]. About a half ofUganda’s total urban population lives in slums. These slums attract high density of low- income earners and or unemployed persons, with low levels of literacy. Evidence notes that almost half percentage of slum dwellers in Kampala are hospitalized due to preventable diseases [20]. Living in an urban informal settlement also increases the risk for stillbirths, early neonatal deaths and perinatal deaths [10, 21]. Therefore it is important to focus on the period around childbirth since most maternal and neonatal deaths cluster around labor and the postpartum period [22, 23]. Despite the adequacy in distribution of health facilities in Kampala both private and public, only 56.4% mothers delivered in public health facilities, 36.5% delivered in private health facilities(not government owned) and 6.7% delivered at home[13]. However, there is no literature on facility deliveries in urban slums of Kampala. Therefore, this study determined the frequency and factors influencing health facility based deliveries among women in urban slums of Kampala Uganda.

## Methods

### Study design and setting

This was a cross sectional study with quantitative methods of data collection. The study was carried out between August and September 2014 in Kampala Central division, Uganda. Kampala is the capital and largest city of Uganda. It is administratively divided into 5 divisions, (Kampala Central, Nakawa, Kawempe, Lubaga and Makindye division), with the total population of 1.5million according to National census of 2014. It accommodates 45% of all urban residents in Uganda and it has a 3.2% population growth rate which has a significant impact on the capacity to plan and deliver services including health services[24]. The city has experienced a population boom in the past years thus compromising the health system. According to the UDHS, the majority of people residing in Kampala are job seekers with a high dependency ratio of 31%. Children under 5 and women of reproductive age (44.6%) comprise nearly half the population of Kampala. These trends have resulted in unprecedented growth of slums and unplanned settlements on the periphery of most towns which is likely to undermine global improvement in maternal and child health if the needs of urban women are not addressed.

The study was conducted in 4 parishes in Kampala Central Division. Kampala city houses the largest urban slums; lying on 14.6sq.km, and administratively divided into 20 Parishes and 135 zones with a population of 90,392. The study area is served by Mulago Hospital, which doubles as the National referral hospital.

### Study population and sampling

The population consisted of women aged between 15–49 years, who gave birth in twelve months preceding the study. Sample size was calculated using Kish Leslieformula, 1995 for cross sectional studies. In total 420 participants were interviewed. Random sampling was used for selection of eligible women who had delivered a live baby in the past one year in Kampala Central Division. At Parish level, 4 parishes were purposively selected because they house urban slums according to classification by Kampala City Council Authority and these parishes had 28 zones altogether. A list of all zones in these parishes was obtained and simple random sampling method was used to select zones to be included in the study. Zone names per parish were written on pieces of paper, folded and put in a box for the researcher to blindly select the required zones. Therefore each zone within a parish had an equal chance of being included in the study. In total 22 zones were selected, two fromKamwokya I Parish, nine fromKamyokya II parish, nine from Kisenyi II parish and two from Kisenyi I parish. The sample size was distributed to the selected zones proportionate to the size of their population. At the zone level, households were selected by systematic random sampling based on sampling frame (list of households) obtained from the chairman of the village (LCI). The sampling interval of the households in each zone was determined by dividing the total number of households to the allocated sample size. If more than one eligible woman were encountered in the household, papers were folded and put down for mothers to choose, in order to determine the woman to be interviewed. When no eligible woman was identified in the selected house hold, the next selected household was the nearest and the same inclusion criteria was applied.

### Ethics

The study was reviewed and approved by the ethics committee at Makerere University School of Public Health Higher Degrees Research and Ethics Committee (HDREC). Permission was got from Local authorities and study subjects were informed about the purpose of the study, their right to refuse and to withdraw. Informed written consent was obtained from each subject before data collection. Confidentiality of the data were kept by avoiding personal identifier and data was kept in a locked room only accessed by the principal investigator.

### Conceptual model

Andersen’s Behavioral Model of Health Services Utilization was used as the conceptual framework[25]. This model has been used widely in both high and middle income countries to understand health services utilization. The model classifies factors that affect health services utilization into three categories: individual, health facility and need factors. The conceptual framework shows how different factors interplay to influence use and non use of health facilities.

### Data collection

Quantitative data were collected usinga structured questionnaire.Interviews were done in the local language (luganda) by trained research assistants. These received training for five days and the content of training included description of study objectives, methods of data collection and sampling techniques. The questionnaire was divided into four major sections; namely; socio-demographics, ANC attendance, labor and delivery and health facility factors. Questionnaires were prepared in English, translated into the local language and back to English to verify if translation reflected the original meaning in English. Research tools were pre-tested from one community in Kampala with similar characteristics as study population. Some questions were refined in a debrief session. Written consent was obtained from respondents before administration of the questionnaire. Interviewstook place on verandas and sometimes inside the house and lasted for an average of 45 minutes. We took measures to ensure that only the respondent was present during the interview.

### Quality control

Each research assistant was supervised once during sessions to observe how the sessions were conducted. Meetings were held to address problems and clarify issues that hampered collection of good data with assistants found to have problems. Checking for accuracy of completed data on questionnaires and notebooks was done at the end of each day of data collection and gaps identified.

### Data management and analysis

Data were double entered into the computer using EPI data which allowed the setting up of proper “skip rules” and “range checks” during data entry, so that errors during data entry were minimized.Data cleaning were done after data entry by running means and checking for out of range values.Data were exported to Stata 12 software for analysis and data exploration was done to visualize the general feature of the data. Simple cross tabulation and chi square test were used for examining the bivariate relationship between the dependent variable and independent variables. The data were expressed in percentages and frequencies (means) at univariable analysis to describe some important characteristics of respondents. Bivariable analysis using logistic regression technique was done to get the crude association between the independent variables and the dependent variable. The strength of association between dependent variable and independent variables (covariates) was expressed using odds ratios (OR).Finally multivariable analysis using backward elimination regression technique was done to evaluate independent effect of each variable on health facility delivery by controlling the effect of others. Socio economic variables such as household income, wealth status, level of poverty, residential neighborhood (socio-structure) were controlled for at multivariate because they are strong confounders of health facility deliveries.

## Results

### Descriptive characteristics of respondents

Of the 423 eligible women identified in selected households, interviews were completed with 420 women, yielding a response rate of 99% for women. The majority of respondents (57.1%) were above 25years and the mean (SD) was 25.8(5.6) years. About 52.3% of respondents and 74% of husbands had post primary education. Majority of respondents 74.5% were married and 56.3% were housewives. Most (58.6%) of the respondents were Baganda, Basoga, Banyankole, Bafumbira, Baganda, Itesot,Acholi, Batooro and other. 72.1% households were headed by husbands and majority of respondents (61.4%) made the final decision on place of delivery. Nearly half of the respondents (51.4%) got health information from other sources (friends, community health workers and relatives) Mothers who get health information from media are 2 times as likely to deliver in health facilities as those who get from other sources (OR = 2.5, 95% CI: 1.6–3.9).(Table 1).

**Table 1:** Socio demographic characteristics of respondents and determinants of facility based delivery among 420 women in urban slums of Kampala.

Characteristics	Frequency (%)	% Utilization		Un adjusted OR (95%C1)	P value
		Yes	No		
**Age**					
15–24	180 (42.9)	68.9	31.1		0.355
≥25	240 (57.1)	64.6	35.4	1.2(0.8–1.8)	
**Mothers education**					
None formal/primary	200 (47.6)	66.5	33.5		0.97
Post Primary	219(52.1)	66.7	33.3	0.9(0.7–1.5)	
Missing	1(0.2)				
**Marital status**					
Un married	107(25.5)	65.4	34.6		0.79
Married	313(74.5)	66.8	33.2	0.9(0.6–1.5)	
**Husbands Education**					
None formal / Primary	87(20.7)	69.0	31.0		0.90
Post Primary	248(59.1)	68.1	31.9	1.04(0.6–1.8)	
Missing	85(20.2)				
**Mothers occupation**					
Not working	236(56.2)	66.5	33.5		0.90
Working	183(43.6)	66.7	33.3	0.9(0.7–1.5)	
Missing	1(0.2)				
**Tribe**					
Baganda	174(41.4)	70.7	29.3		0.12
Other tribes*	246(58.6)	63.4	36.6	1.2(1.0–1.6)	
**House hold head**					
Wife/Other	114(27.1)	63.2	36.8		0.40
Husband	294(70.0)	67.3	32.7	0.8(0.5–1.3)	
Missing	12(2.9)				
**Final decision on place of delivery**					
Self	258(61.4)	64.7	35.3		0.40
Husband	160(38.1)	68.7	31.3	0.8(0.5–1.3)	
Missing	2(0.5)				
**Source of health information**					
Other sources**	216(51.4)	76.8	23.2		**<0.001**
Media	204(48.6)	55.4	44.6	2.7(1.8–4.1)	

Other tribes

*Basoga, Banyankole, BafumbiraBaganda, Itesot,Acholi, Batooro. Other source

** friends, CHWs and relatives

### ANC attendance and need based factors associated with health facility delivery

About half (55.5%) of respondents had 2 or more children, and 50.1% had pregnancy complications in the most recent pregnancy. Nearly a half (50.4%) started labor during the day and 93.6% had prepared for birth. A large percentage of respondent’s 95.7% attended ANC at least once and 65.8% at least had 4 ANC visits. Majority of women (60.9%) attended their first ANC in the first 3 months of pregnancy. Mothers who attended ANC less than 4 times were less likely to go for health facility delivery than those who attended at least 4 times (OR = 0.6 95% CI 0.3–0.9); and mothers who had their first ANC visit in the 2 or 3^rd^ trimesters of pregnancy were less likely to use health facilities for delivery compared to those who had their ANC in the 1^st^ trimester (OR 0.5 95% CI 0.3–0.8). (Table 2)

**Table 2:** ANC attendance and need based factors associated with health facility delivery.

Characteristics	Frequency (%)	% Utilization		Un adjusted OR (95%C1)	P value
		Yes	No		
**Parity**					
1	187(44.5)	65.8	34.2		
≥2	233(55.5)	66.9	33.1	0.9(0.6–1.4)	0.80
**Pregnancy complications**					
Yes	210(50.0)	61.9	38.1		
No	209(49.7)	71.3	28.1	0.6(0.4–0.9)	0.04
Missing	1(0.3)				
**Time labor started**					
Night	208(49.5)	65.4	34.6		
Day	211(50.2)	67.3	32.7	0.9(0.6–1.4)	0.67
Missing	1(0.3)				
**ANC attendance**					
Yes	402(95.7)	66.9	55.6		
No	18(4.30)	33.1	44.4	1.6(0.6–4.2)	0.32
**Frequency of ANC**					
At least 4 times	138(32.9)	63.0	37.0		
Less than 4 times	266(63.3)	68.4	31.6	0.7(0.5–1.2)	0.27
Missing	16(3.8)				
**Timing of 1**^**st**^ **ANC Visit**					
≤1^st^ trimester	158(37.6)	60.8	39.2		
≥2^nd^ trimester	246(58.6)	70.7	29.3	0.6(0.4–0.9)	0.04
Missing	16(3.8)				
**Birth preparedness**					
Yes	392(93.3)	67.4	32.6		
No	27(6.4)	51.8	48.2	1.9(0.8–4.2)	0.10
Missing	1(0.3)				

### Health system factors associated with health facility delivery among study participants

Majority of respondents (65.7%) took 30 minutes or less to reach a health facility, 73.6% were satisfied with the quality of care offered and 58.3% said the services were not costly. Mothers who perceived the cost of care to be low were more likely to use health facilities at delivery compared to those who perceived it to be high (OR = 1.5 95% CI 0.9–2.3).Table 3

**Table 3:** Health system factors associated with health facility delivery among study participants.

Characteristics	Frequency (%)	% Utilization		Un adjusted OR (95%C1)	P Value
		Yes	No		
**Distance (min)**					
> 30 min	272(64.8)	68.7	31.3		
<30 min	142(33.8)	64.1	35.9	1.2(0.8–1.9)	0.34
Missing	6(1.4)				
**Perceived quality of care**					
Good	304(72.4)	69.4	30.6		
Not good	109(25.9)	61.5	38.5	1.4(0.9–2.2)	0.13
Missing	7(1.7)				
**Perceived cost of services**					
High	175(41.7)	73	27		
Low	245(58.3)	63.9	36.1	1.5(0.9–2.3)	0.05

Significant variables at bivariate, variables with biological plausibility and significant variable in literature were included in the analysis. They included source of health information, times attended ANC, timing of first ANC, birth preparedness, quality of care and cost of care. The table below shows the adjusted OR and 95% CI derived from logistic regression. (Table 4).

**Table 4:** Logistic regression analysis of factors that predicted health facility delivery among women in urban slums of Kampala.

Characteristic	Crude OR	Adjusted OR	P Value
**House hold head**			
Wife/other			
Husband	0.8(0.5–1.3)	0.7(0.5–1.2)	0.232
**Source of health information**			
Other sources			
Media	2.7(1.8–4.1)	2.5(1.6–3.9)	**<0.001**
**Frequency of ANC**			
At least 4 times			
Less than 4 times	0.7(0.5–1.2)	0.6(0.3–0.9)	**0.024**
**Timing of 1**^**st**^ **ANC**			
1^st^ trimester			
≥ 2^nd^ trimester	0.6(0.4–0.9)	0.5(0.3–0.8)	**0.005**
**Perceived cost of services**			
High			
Low	1.5(0.9–2.3)	0.7(0.5–1.1)	0.172

From the table above, Mothers who get health information from media are 2 times as likely to deliver in health facilities as those who do not get information from the media (OR = 2.5, 95% CI: 1.6–3.9). Mothers who attended ANC less than 4 times are less likely to go for health facility delivery than those who attended at least 4 times (OR = 0.6 95% CI 0.3–0.9). And mothers who had their ANC visit in the 2 and 3^rd^ trimesters of pregnancy are less likely to use health facilities at delivery compared to those who had their ANC in the 1^st^ trimester (OR 0.5 95% CI 0.3–0.8).

## Discussion

The study demonstrates that only two thirds of slum dwellers utilized health facilities for delivery care which is lower than the national average for Uganda.These findings are in close agreement with the recent UDHS report and findings from other studies carried out among urban poor in India and Nigeria[13, 26, 27].Despite the high number of pregnant women that attended ANC in this study (95.7%), a third(3%) still had a home delivery. This finding is not different from that of other studies conducted in Uganda where majority of mothers(95%) attended ANC but few delivered in health facilities[16, 28]. Factors associated with a non-health facility delivery included starting ANC late,attendingless than 4 ANC visits and poor media exposure. Several explanations have been put forward to account for the effects of antenatal care on the subsequent decision to give birth in a medically qualified environment. The main reasons mothers give for attending ANC in big numbers are[29], for the well-being of the fetus, knowing their HIV/STI status so that they protect the fetus, as well as getting the card which will act as an entry ticket to formal health care services incase complications occur during birth. ANC is more effective in preventing adverse pregnancy outcomes when it is sought early in pregnancy and continued throughout pregnancy[29, 30]. Only 39% of the pregnant women in this study made their first ANC visits before the fourth month of pregnancy. This indicates that, women in the study area started ANC at relatively late stage of pregnancy, thus limiting the quality of care provided and hampers the delivery of effective antenatal care screening and treatment programs, potentially contributing to the high maternal morbidity and mortality[31].

In this study, we found that the key factors that were significant in determining health facility births, among urban poor women were, the main source of health information, times attended ANC and timing of first ANC visit.Main source of health information among the respondents was found to be a key factor in determining health facility births (OR = 2.5 95% CI 1.6–3.9). Mothers who got information from media were more likely to give birth in health facilities than mothers who did not get information from the media. This finding is in line with other studies done in Ethiopia and India[30, 32] which showed that Women who had exposure to Radio or TV were almost three times more likely to attend delivery care than those without exposure to media (OR = 2.998). The possible explanation for this is that exposure to mass media promotes health-related behaviors including reproductive health services that could facilitate behavioral changes [33].

The findings report that 37.6% of women started their ANC in the 1^st^ trimester. This is consistent with a report from Kenya where only 36% of the women started attending ANC in the first and second trimesters[34]. Late ANC attendance may reduce women’s chances of benefiting fully from preventive strategies, such as iron and folic acid supplementation, treatment of infections, and intermittent preventive treatment for malaria in pregnancy. Early ANC attendance provides opportunities for health education, recommended place of delivery and information on the status of their pregnancy, which in turn informs their decisions on where to deliver.

The study also shows that frequency of ANC attendance predicts facility based delivery. The WHO recommends that a pregnant mother without any complications should have at least four ANC visits to provide sufficient information of her own health and developing fetus. Early booking of antenatal care is important as it provides health workers with the opportunity of early detection of maternal problems and corrective measure taken immediately to get rid of them for benefit of mother and fetus. In this study 95.7% women attended ANC at least once, but 65.8% had at least 4 visits. After adjusting for other factors at multivariable analysis, the relationship between place of delivery and frequency of antenatal visit was significant as those who attended less than four visits were less likely to deliver in health facility compared to those who attended ANC at least four times OR = 0.6. The findings are similar to previous findings from Rwanda, Uganda and Combodia; where there is increased likelihood for health facility deliveries among women who attended ANC more than four times than those women that attended less than four times.[35–37]. This might be due to the fact that during ANC visits, especially if started early, women are provided with health education and information about the benefits of delivering in health facility.

However, it is worth noting that these findings contrast findings conducted among urban poor in Mumbai-India and Nigeria where over half delivered outside hospital facilities and 81.8% of those deliveries were not attended by a skilled health worker [38, 39].

### Study strength and weaknesses

This study has several strengths but also some weaknesses. The strength include it being one of the first studies in Uganda to assess access to delivery care in urban settings in Uganda and with a large sample size.The weaknesses included, the inclusion criteria used could have introduced selection bias because only mothers who had a live baby were interviewed thus excluding mothers who had lost their newborns at birth or whose newborns died before the time of interviews. This may have inflated the frequency of health facility deliveries as perinatal deaths are more likely to happen in home deliveries.

The major policy and program implications of this study is the fact that attention should be paid to urban poor in the same way as it is paid to those in rural communities. Many programs by pass urban settings in the mistaken belief that those in the urban settings have a good access to care or implementing in rural settings is assumed to be following the principles of primary health care. People in slums may have physical access as shown in this study, but lack affordability and may actually be less informed. Currently in Uganda the government is scaling up use of vouchers, village health teams to link pregnant women to health facilities, building and reconstruction of maternal wards and other forms of result based financing. Howeverthese plans are targeting people living in rural districts, thus leaving out the urban poor despite the increasing population in such settings. Attention to the health of urban poor will lead toincreased access thus improving the health status of the whole population.

## Conclusion

Despite good physical access, a third of mothers did not deliver in health facilities. Increasing health facility births among the slum dwellers can be improved through interventions geared at increased awareness, encouraging and educating mothers on thebenefits of attending ANC at least four times and starting ANC in the first trimester of pregnancy.

## Supporting information

S1 File Questionaire. (XLSX)

S2 File Raw data -determinants of health facility delivery. (DOCX)
