# Supplementary material for: Determinants of facility based–deliveries among urban slum dwellers of Kampala, Uganda
Source: PLoS One. 2019 Apr 18;14(4):e0214995. doi: 10.1371/journal.pone.0214995 (PMC6472760; doi:10.1371/journal.pone.0214995)
Supplement: S2 File — (DOCX) [file pone.0214995.s002.docx]

### Questionnaire.

**Screening question.** Ask if there is any woman in the house hold who delivered within the last 1 year. If yes continue with the interview, if no thank her and go to another house hold.

Interview No……………………………

Name of the zone………………………

Date of the interview…………………….

|  | How old are you? …………………… |  |
| --- | --- | --- |
|  | How many years you spent in school?...................... |  |
|  | Name of the baby between 1 – 12 months…………… |  |
|  | Age of Baby in Months .................. |  |
|  | Mode of delivery   1. Normal 2. Caesarian 3. Breech 4. Other specify…………………….. |  |
|  | What was the outcome of labor?   1. Live baby 2. Still birth 3. Early neonatal death |  |
|  | Number of pregnancies mother has had ………………. |  |
|  | Number of children mother has had ……………………. |  |
|  | Number of children who are alive ………..………………. |  |
|  | What is your marital status?   1. Single 2. Married 3. Divorced/ Separated 4. Widowed 5. Other specify |  |
|  | To which tribe do you belong? ……………………. |  |
|  | What is your occupation?   1. House wife 2. Casual laborer 3. Vendor 4. Salaried worker 5. Self employed /business 6. Other specify……………. |  |
|  | What is your husband’s Occupation?   1. Casual laborer 2. Mechanic 3. Vendor 4. Salaried worker 5. Self employed /business 6. Other specify ………………… |  |
|  | How many people are living with you in your house hold? …… |  |
|  | Who is the head of your house hold?   1. Yourself 2. Your husband 3. Other specify ……………….. |  |
|  | What is your main source of information about health issues.   1. Radio/television 2. Billboards 3. Health worker 4. Community campaign 5. Friends/relatives 6. Other (specify |  |

**ANC ATTENDANCE**

| 1. 2 | During the last pregnancy, did you attend ANC ?   1. Yes 2. No |  |
| --- | --- | --- |
|  | If yes, how many times did you attend in the last pregnancy? …………… |  |
|  | How many months was the pregnancy when you attended your first ANC? ............. |  |
|  | Whom did you see for ANC the first time?   1. Doctor/medical assistant/clinical officer 2. Midwife /nurse 3. Nursing assistant 4. TBA 5. VHT/CHW 6. Other Specify |  |
|  | How many times do you think are appropriate? |  |
|  | Did you prepare for child birth?   1. Yes 2. No |  |
|  | During the last pregnancy, did any healthcare provider (nurse/midwife/TBA/VHT/CHW or other) visit you at home to provide care or advice?   1. Yes 2. No |  |

**LABOUR AND DELIVERY**

| 1. 3 | When did the labor start?   1. At night 2. During the day |  |
| --- | --- | --- |
|  | If (1) above, did it influence the choice of place of delivery?   1. Yes 2. No |  |
|  | If (1) above, where did you deliver your baby?   1. Own home 2. TBA’s home 3. Health centre 4. Private clinic 5. Hospital 6. Other specify ………………… |  |
|  | Who decided on the place of your last delivery?   1. Self 2. Husband 3. Mother inlaw 4. Other (specify) |  |
|  | What were the reasons for the above choice?(tick all that apply)   1. Sudden onset of labor 2. Offer friendly services 3. Less cost of services 4. Easy to access(distance) 5. Good attitude of health workers/ TBA 6. Advice from a health worker 7. Advice from a friend 8. Complications /Abnormal labor 9. Other (specify)……………………. |  |
|  | For those that did not deliver at a health facility, Why didn’t you deliver in a health facility? (tick all that apply)   1. Cost of treatment too much 2. Facility not open 3. Facility too far 4. No transport 5. No money for transport 6. Poor services 7. No female providers 8. Didn’t have required supplies 9. Other specify |  |
|  | Was it the place you intended to deliver from?   1. Yes   No |  |
|  | If no, where had you intended to deliver from?   1. Own home 2. TBA’s home 3. Health facility 4. Other specify ………………… |  |
|  | Who assisted you during delivery?   1. Doctor/medical assistant/clinical officer 2. Midwife /nurse 3. Nursing assistant 4. TBA 5. VHT/CHW 6. Other Specify |  |

**HEALTH FACILITY FACTORS**

| 1. 4 | On average how far is the health facility from your home?   1. Less than one hour 2. 1-2 hours |  |
| --- | --- | --- |
|  | Are you satisfied with the services provided at your health facility?   1. Yes 2. No |  |
|  | If no, what makes you unsatisfied with the services provided at your facilities? (Tick all that apply)   1. No drugs and supplies 2. Bad behavior of health workers 3. Lack of privacy 4. Not allowed to ask questions 5. health workers not help full 6. place not clean 7. Other specify ………………. |  |
|  | Is there any reward provided for mothers who deliver in health facilities?   1. Yes 2. No 3. Don’t know |  |
|  | If yes, what reward is provided? ................................. |  |
|  | Is there any payment of any form given to health facilities after assisting in delivery?   1. Yes 2. No 3. Don’t know |  |
|  | If yes which form of payment is given? … |  |
|  | Is there any traditional habit in your community that should be done before delivery? (Please mention) …………………………………………….. |  |
|  | Is there any traditional belief that prevents women to deliver in health facilities in this community? (mention it) ……………………….. |  |
|  | What is your recommendation for improving services in your health facility? ……………………………………………………………………… |  |

**THANK YOU FOR YOUR TIME AND PARTICIPATION**
